# Non‐verbal effecting – animal research sheds light on human emotion communication

**DOI:** 10.1111/brv.13140

**Published:** 2024-09-11

**Authors:** Annett Schirmer, Ilona Croy, Katja Liebal, Stefan R. Schweinberger

**Affiliations:** ^1^ Department of Psychology Innsbruck University Universitaetsstrasse 5‐7 Innsbruck 6020 Austria; ^2^ Department of Psychology Friedrich Schiller University Jena Am Steiger 3 Jena 07743 Germany; ^3^ German Center for Mental Health (DZPG) Partner Site Halle‐Jena‐Magdeburg Virchowweg 23 Berlin 10117 Germany; ^4^ Institute of Biology Leipzig University Talstraße 33 Leipzig 04103 Germany

**Keywords:** facial expressions, emotion recognition, non‐verbal communication, body language, affective

## Abstract

Cracking the non‐verbal “code” of human emotions has been a chief interest of generations of scientists. Yet, despite much effort, a dictionary that clearly maps non‐verbal behaviours onto *meaning* remains elusive. We suggest this is due to an over‐reliance on language‐related concepts and an under‐appreciation of the evolutionary context in which a given non‐verbal behaviour emerged. Indeed, work in other species emphasizes non‐verbal *effects* (e.g. affiliation) rather than meaning (e.g. happiness) and differentiates between signals, for which communication benefits both sender and receiver, and cues, for which communication does not benefit senders. Against this backdrop, we develop a “non‐verbal effecting” perspective for human research. This perspective extends the typical focus on facial expressions to a broadcasting of multisensory signals and cues that emerge from both social and non‐social emotions. Moreover, it emphasizes the consequences or effects that signals and cues have for individuals and their social interactions. We believe that re‐directing our attention from verbal emotion labels to non‐verbal effects is a necessary step to comprehend scientifically how humans share what they feel.

## I. INTRODUCTION

What's a wink and a smile? In both popular language and science, they are non‐verbal behaviours individuals use to convey an affective message to other individuals. From the ancient philosophers to modern empiricists, there has been a keen interest in developing a dictionary that maps a given behaviour onto an intended meaning. However, even after much dedicated research, such a dictionary remains elusive. Although available findings sketch out basic correspondences, they also characterize non‐verbal emotion communication as complex and often ambiguous. Here, we seek to address this problem by drawing on findings from non‐human animals and the manner in which communication is studied and conceptualized there. We apply the non‐human approach to humans and formulate a novel framework with the goal to understand non‐verbal expressions of emotions (NVEEs) better in our species.

## II. MODALITIES OF HUMAN NON‐VERBAL EXPRESSIONS OF EMOTIONS

**Fig. 1:**
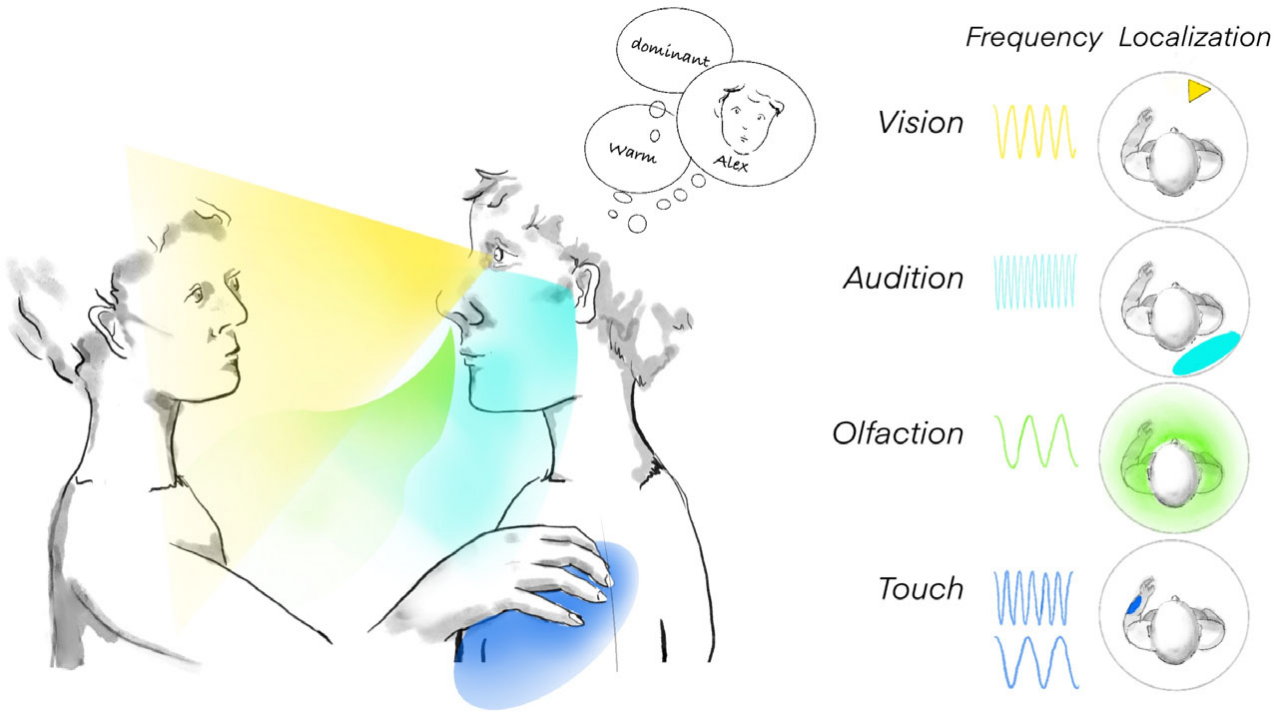
Non‐verbal sensory modalities. We perceive non‐verbal activity mainly *via* vision, audition, olfaction, and touch. These senses differ in their temporal and spatial precision. While the temporal resolution of auditory representations is high, it is low for olfactory representations. Moreover, olfactory representations cannot be localized without the aid of other senses (Croy *et al*., 2014) and can only be detected at close distance. In comparison, the visual sense allows precise localization and operates over longer distances. Tactile sensing relies on different nerve fibres varying in their temporal and spatial resolution. Touch is only evoked by objects that come into physical contact with the individual. Humans also perceive each other *via* the gustatory channel. However, social situations in which licking and tasting play a role are quite rare and mainly relate to sexual interactions.

Although emotions are a ubiquitous human experience, their definition has been surprisingly difficult and much debated. For example, whether they comprise discrete categories, what those categories might be, and what cognitive processes are relevant remain open questions. Yet, some basic ideas repeat across theoretical perspectives, including that emotions arise from specific events as a result of event appraisals that can be automatic and potentially biologically prepared (Schirmer, 2014; Scherer & Moors, 2019). Additionally, there is some consensus that emotions motivate behaviour and are thus drivers for non‐verbal communication (Ekman, 1992; Panksepp, 2004; Dael, Mortillaro & Scherer, 2012; Keltner *et al*., 2019; Freeberg *et al*., 2021). Emotions are thought to lead to broadband changes in physiological, mental, and motor processes that ready the body for action and that are often perceptible to others. Although these changes do not neatly map onto putative emotion categories (Barrett *et al*., 2019), they nevertheless provide information about senders. Research tackling their information value has traditionally pursued them separately for the different human sensory modalities (Fig. 1).

**Fig. 2:**
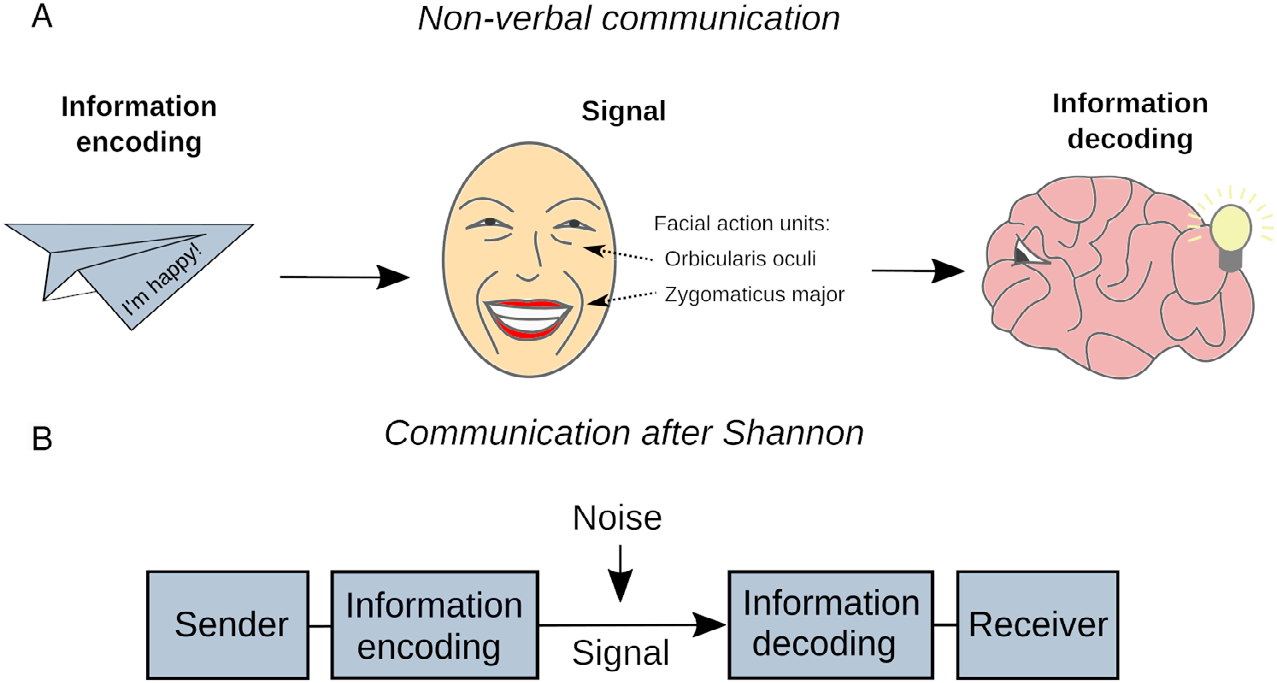
Traditional communication models. (A) Traditional thinking holds that non‐verbal activity is a signal representing a communicative intent *via* a bodily code. Receivers must decode or translate the signal in order to understand its meaning. (B) Shannon developed a mathematical communication model to addresses the technical challenges of long‐distance communication. His simplified model has been used to formalize non‐verbal processes.

Vision is often considered the most important sense in this context. It enables the perception of dynamic facial changes, but also other bodily motions such as head movements, hand gestures, or gait. To date, research has emphasized faces with the goal of relating these to an individual's communicative intent (Berger *et al*., 2020). A popular, albeit controversial, approach is Ekman's facial action coding system, which defines action units associated with one or multiple muscles and maps them to specific emotions (Ekman & Friesen, 1978). For example, Ekman considers activity in the orbicularis oculi (i.e. circular muscle around each eye) and the zygomaticus major (i.e. muscle at lip corners) as an expression of happiness (Fig. 2A).

Audition allows us to perceive the sounds produced by others. This concerns vocalizations such as spoken language and its accompanying prosody as well as non‐speech exclamations like laughter. Voices have been pursued analogously to faces by considering their constituents, in this case acoustic parameters like fundamental frequency, tempo, or intensity. One example is the Brunswikian lens model (Brunswik, 1952) as adapted by Scherer and colleagues (Scherer, 1986; Banse & Scherer, 1996), which holds that unobservable feeling states shape vocalizations by changing acoustic parameters in a finger‐print manner. In listeners, these parameters then elicit specific subjective percepts such that vocalizations may appear as high‐pitched, fast or loud. When integrated, these percepts enable inferences about a vocalizer's emotions (Bänziger, Hosoya & Scherer, 2015), for example, that they feel happy. Work applying a Brunswikian approach found unique combinations of parameters and percepts that carry information about specific emotion families (Bänziger *et al*., 2015).

Among the somatosensory modalities, the tactile sense is of particular relevance for NVEE. Through a variety of specially tuned mechanoreceptors, it represents all manners in which humans physically interact (McGlone, Wessberg & Olausson, 2014). Building upon earlier facial and vocal studies, relevant work on touch required an individual to contact another so as to convey a specific emotion. Results revealed fairly stereotyped tactile actions that receivers interpreted better than would be expected by chance (Hertenstein *et al*., 2006, 2009). Happiness, for example, was most frequently expressed by shaking or swinging another's arm or other body part.

Last, there are the chemical senses, which rarely have been explored to date. Arguably, we are more likely to smell than taste each other and it is hence not surprising that research has focused on the olfactory pathways, showing for instance that mothers derive happiness from their infant's body odour (Schäfer *et al*., 2020). Body odours are altered by changes in metabolism leading to a change in the composition of volatiles. Their release in the context of sweating is enhanced by sympathetic activation and thus affected by transient states. Examples include inflammation (Olsson *et al*., 2014), anxiety (Prehn *et al*., 2006), or aggression (Pause, Storch & Lübke, 2020), which each elicit olfactory traces that can be discriminated from neutral odours.

In sum, emotions shape human non‐verbal behaviour along multiple dimensions. For research purposes, these dimensions have been mapped onto the different sensory modalities by which humans perceive their environment.

## III. TRADITIONAL PERSPECTIVE ON HUMAN NON‐VERBAL EXPRESSIONS OF EMOTIONS

The study of human NVEEs has been dominated by the idea that one individual sends an emotional message to another, receiving, individual. This idea is reflected in the classic sender–signal–receiver model (Fig. 2B), first explicitly formulated by Claude Shannon in the context of long‐distance signal transmission for applications such as the telephone (Shannon, 1948). In simple terms, this model involves an encoder that turns information into a transmittable signal, a channel for relaying the signal, and a decoder that reconstructs the initial information for a receiver. Within this framework, a person feeling jovial might encode this feeling into a wink and a smile, which are signals conveyed *via* a visual channel. An interaction partner acts as a receiver. The receiver's neural processes decode the signals, thus allowing for the jovial feeling to be understood. Here, winking and smiling, just like words, represent a code associated with a particular meaning.

Unfortunately, the sender–signal–receiver model has had only partial success. While studies found that the explicit human recognition of NVEEs consistently exceeds chance, it arguably does not exceed it by much, especially under rigorous testing conditions. Indeed, how emotions are encoded and decoded appears to differ among cultures (Elfenbein *et al*., 2007; Sauter *et al*., 2010; Jack *et al*., 2012; Gendron *et al*., 2014; Laukka & Elfenbein, 2021), among individuals within a culture (Elfenbein & Ambady, 2002; Laukka *et al*., 2013; Schirmer, 2013), and even within individuals (Schirmer, Kotz & Friederici, 2005; Skuk & Schweinberger, 2013) thus implying that emotions are not communicated *via* a simple, unambiguous code.

Research examining NVEEs has treated this issue methodologically rather than conceptually by employing a multi‐step procedure aimed at optimizing expressive stimuli, although in a manner that compromises stimulus naturalness or validity [for recent alternative approaches see Schaefer *et al*. (2010) and von Eiff, Kauk & Schweinberger (2023)]. This procedure was first established in the context of human face research (for a review see Bänziger, Mortillaro & Scherer, 2012; Dawel *et al*., 2022) and has subsequently been adopted for other modalities (Pell, 2002, 2005; Schirmer *et al*., 2004; Ferdenzi *et al*., 2011; Bänziger *et al*., 2012). Typically, lay or professional actors pose emotions based on muscle‐directed instructions (Ekman & Friesen, 1976), by being given a target emotion (Lundqvist, Flykt & Öhman, 1998; Tottenham *et al*., 2009), or by interpreting an emotion scenario (Bänziger *et al*., 2012). The produced stimuli are then evaluated for their accuracy before being used in research. In the context of faces, this has been done by comparing the produced expression against a target prototype (Ekman & Friesen, 1976; Simon *et al*., 2008). However, a more common approach relies on naive raters or judges who categorize stimuli based on a list of emotion terms that apart from the target emotions typically includes “neutral” or “other”. Only stimuli that cross a certain recognition threshold (e.g. 80% of the participants recognized the intended emotion) are then selected for the experiment (Pell, 2002, 2005; Schirmer *et al*., 2004; Bänziger *et al*., 2012).

Despite these efforts, however, the mean recognition accuracy in actual experiments is typically far from perfect (Barrett *et al*., 2019). For example, in early work by Ekman & Friesen (1971), participants had to identify the correct expression label among two or three alternatives. With the binary task, performance ranged from 76 to 100%, whereas for the tertiary task it ranged from 28 to 100% with the lowest accuracy for fear faces when labels included fear, surprise and sadness. More recent work found similar results. In a more challenging set‐up with six emotion faces for which participants had to select one of seven response options (six emotion labels plus “other”), accuracy ranged from 60 to 97% (Chóliz & Fernández‐Abascal, 2012). Notably, emotion recognition from other modalities tends to be worse. For instance, when compared with visual displays, emotional vocalizations elicited approximately 20% lower accuracy (Bänziger, Grandjean & Scherer, 2009) and body odours were correctly categorized as “frightened” in no more than 58% of cases when raters had three alternative choices (Ackerl, Atzmueller & Grammer, 2002). Touch, when communicating one of 12 emotions and judged using 13 response options (i.e. 12 emotion labels plus “other”), was found to have an accuracy ranging from about 18 to 83% (Hertenstein *et al*., 2006). As detailed elsewhere, recognition rates in categorical mapping tasks overestimate true recognition, which declines even further when task formats are less constraining (Barrett *et al*., 2019).

Some attempts have been made to examine the recognition of spontaneous NVEEs. For example, audio recordings from field settings or emotion‐provoking laboratory settings have been presented to naive listeners who attempted to classify them (Juslin *et al*., 2021; Szameitat, Szameitat & Wildgruber, 2022). Additionally, work examining olfactory communication has, by necessity, elicited emotions in, for example, sweat or tear donors and then exposed receivers to a range of the obtained samples. While this work has focused largely on automatic and potentially unconscious responses like emotional contagion (de Groot & Smeets, 2017), it also revealed some evidence that receivers can explicitly identify a sender's affective state (Chen & Haviland‐Jones, 2000). Notably, however, the emotions of such natural, spontaneously produced expressions are significantly more challenging to identify than those of posed expressions by both humans and computer algorithms, ostensibly because their category boundaries are less clear (Krumhuber *et al*., 2021).

**Fig. 3:**
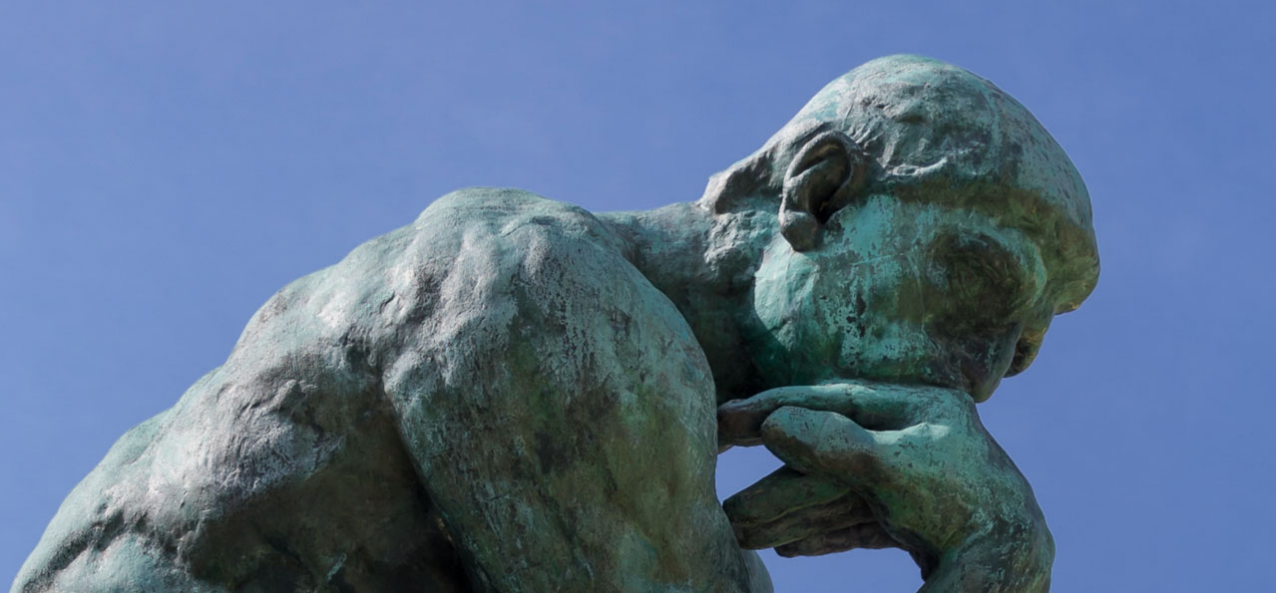
Auguste Rodin's *Thinker* (Wiki, Creative Commons Attribution 2.0 Generic license) illustrates non‐communicative non‐verbal activity (e.g. furrowed brows, self‐touch, fixed gaze, bent posture).

The poor performance of both posed and spontaneous NVEEs agrees with observations of multi‐mapping. One such observation is that different and seemingly incongruous non‐verbal behaviours may be evoked by the same emotion. One example is a joyful state accompanied by both laughter and sobbing. Another example is a fearful state, which may provoke submissive as well as aggressive behaviours. The latter fear responses have been explained by the cascade defence model implying that emotions adaptively regulate physiological and motor activity based on situational constraints (Lang, Davis & Öhman, 2000). Proximal when compared to distal threat is more likely to prompt a fight reaction. Additionally, the same non‐verbal behaviour may accompany different and seemingly incongruous emotions. The smile takes a variety of forms and is exhibited in a variety of situations, including when we are happy, proud, amused, scheming, sad, or fearful. Indeed, it has been recognized as the most ambiguous facial expression (LaFrance, Hecht & Paluck, 2003). There are other similarly ambiguous examples such as the frown. Although central to the stereotypical anger display, a frown also indexes effortful processing independently of emotion (Berger *et al*., 2020) as observable in Rodin's *Thinker* (Fig. 3).

To summarize, the study of human non‐verbal emotion communication has been guided by a sender–signal–receiver model and, to enhance recognition performance, often relied on posed displays that were mapped onto circumscribed emotion categories. This work has failed to explain the substantial variability in non‐verbal emotion communication and to elucidate its underlying mechanisms.

## IV. WHAT IS WRONG WITH THE TRADITIONAL HUMAN PERSPECTIVE?

The sender–signal–receiver model usefully guided early research on non‐verbal emotion communication. However, we argue that its human‐centric focus is precluding further progress due to an over‐reliance on what many consider special human abilities. This includes drawing parallels with language when conceptualizing and discussing non‐verbal processes. Like language, non‐verbal behaviours are thought to refer to something that individuals are motivated to communicate and this is reflected in both popular and scientific terminology, which is dominated by language‐relevant descriptors (e.g. “body language”, or “non‐verbal code”). Yet, although non‐verbal behaviours can be referential, they do not need to be. Furthermore, NVEE, like language, is often placed in the broader context of human cognition. Although the modern understanding of cognition entails processes that are automatic, a cognitive lens may nevertheless bias us towards conscious, effortful, and analytical mechanisms when studying NVEEs. Lastly, beginning with Darwin's study of expressions of emotions (Darwin, 1872), the study of human NVEEs has focused on the face at the expense of other non‐verbal channels. As the study of different channels may yield different insights, results may align differently with a sender–signal–receiver model. Indeed, compared to facial emotion recognition, emotion recognition from auditory, tactile and olfactory channels is poorer and the idea that they comprise neat expressive categories or codes is even less convincing.

**Fig. 4:**
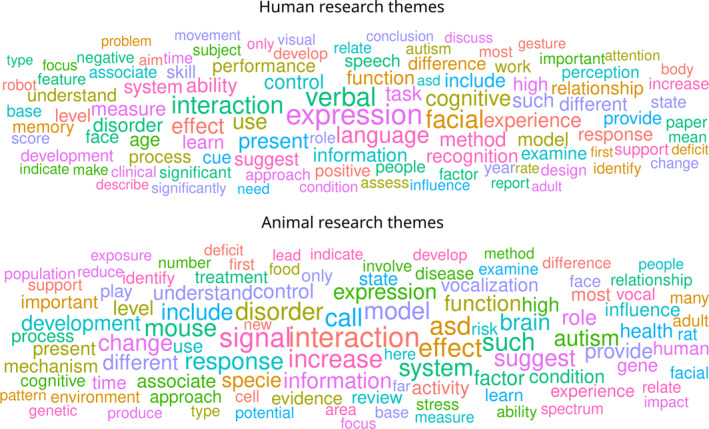
Frequency‐scaled representation of lemmas in publication abstracts selected in the context of human (*Scopus* search “TITLE‐ABS‐KEY (emotion* OR affect*) AND TITLE‐ABS‐KEY (nonverbal OR non‐verbal) AND NOT TITLE‐ABS‐KEY (artificial AND intelligence)”) and non‐human research (*Scopus* search “TITLE‐ABS‐KEY (emotion* OR affect*) TITLE‐ABS‐KEY (communication AND social) AND TITLE‐ABS‐KEY (nonhuman OR non‐human OR animal)”). The more frequent a lemma is, the larger its font. Themes that dominate human but not animal research include “verbal”, “facial”, “language” and “cognitive”.

To corroborate and illustrate these points, we sought to identify dominant themes in human non‐verbal research *via* an automated literature search (see online Supporting Information, Appendix S1; Fig. 4). Our search identified, from the *Scopus* database, 10,800 publication abstracts dealing with human NVEEs. We subjected these abstracts to a text analysis routine that returned unique lemmas and counted their respective frequencies across the abstracts. Figure 4 shows the most frequent 100 lemmas with font size indexing frequency. The term “expression” was most frequent, followed by “verbal” and “facial”; “language” and “cognitive” were in sixth and seventh place, respectively. Thus, these results match our perceptions of the human literature as detailed above.

Interestingly, how NVEE research is conceptualized or framed differs when the subjects are non‐human animals. Here, an analogous analysis of 2317 published abstracts identified “interaction” as the most frequent term followed by “signal” and “effect”. While “call”, a term referencing interest in auditory communication, was in sixth position, the terms “verbal”, “facial” and “cognitive” were not among the top 50 terms. This implies meaningful variation in the underlying conceptual approach. Indeed, language‐related concepts, cognitive functions, and facial displays are less pervasive in the discussion of non‐human when compared with human non‐verbal emotion communication.

Taking a step back from language, cognition and faces and making fewer assumptions about the meaning and complexity of non‐verbal processes may be helpful by prompting simpler, more parsimonious mechanistic explanations. One such explanation, as proposed by Darwin (1872), is that some non‐verbal behaviours evolved, not to relay information, but as tools or effectors that regulate one's own state either directly, or indirectly by changing the behaviour of an interaction partner. A direct and thus self‐regulatory example is a startled animal closing its eyes not to relay its shock but to protect its eyes from injury. An example of indirect regulation is an animal raising its fur during aggressive encounters not to relay readiness to attack but to appear bigger and thus more intimidating to an opponent.

A tool‐ or effect‐based mechanism has already entered the discussion of human NVEE (Susskind *et al*., 2008; Crivelli, Carrera & Fernández‐Dols, 2015; Scarantino, Hareli & Hess, 2022). In particular, Fridlund developed a framework called the behavioural ecology view of facial displays (BECV) in which he contrasted traditional ideas about the meaning of facial expressions with an effect‐based account (Fridlund, 1994; Crivelli & Fridlund, 2018). For example, the BECV assumes that smiles prompt “a partner to play or affiliate” rather than to express happiness and that frowns prompt “a partner to submit” rather than to convey anger. Notably, effects associated with these and other non‐verbal displays are concerned strictly with the face and rest on the assumption that displays are always socially motivated and thus intended for an audience.

We agree with the BECV that our understanding of NVEE can be facilitated by insights from non‐human communication research. Yet, we believe a broader perspective going beyond both the face and social emotions is needed. Here, we seek to develop such a perspective by mapping ideas and terminology derived from both human and non‐human research, by re‐considering the relationship between emotions and non‐verbal behaviours, and by formulating basic principles of what we call “non‐verbal effecting”.

Taken together, the popular view of human NVEE is compromised by an exaggerated focus on language‐like processes, cognitive effort, and faces. Past attempts to incorporate insights from non‐human animal research have been limited.

## V. CONCEPTUALIZING COMMUNICATION IN NON‐HUMAN ANIMALS

Because emotions serve important survival functions, they likely exist not only in humans but also in non‐human animals. Yet, because non‐human animals differ in their behavioural repertoires and survival tasks, they undoubtedly differ in the nature of their emotions (Bliss‐Moreau, 2017; Paul & Mendl, 2018). Indeed, some theoretical perspectives go as far as to postulate that non‐human species experience only basic affective states that lack concrete subjective feelings and that therefore do not qualify as emotions (Barrett *et al*., 2007). However, given the lack of consensus about what an emotion is and what defines its subjective feeling aspects, we consider this differentiation unhelpful. In what follows, we use the term emotion to reference the same bio‐behavioural system across species and examine its relevance for non‐human animal communication.

In the context of non‐human animal research, the term “communication” is used to describe the process by which a change in one individual's behaviour causes a change in another individual, mediated by either signals or cues (Freeberg *et al*., 2021). Signals are non‐verbal expressions, which evolved for their effect on others (Krebs & Davies, 1993), such as mating displays that index a sender's fitness or alarm calls that warn about predatory threat (Smith & Harper, 1995). They benefit senders indirectly by changing a receiver's behaviour. Cues, on the other hand, are any animal activity (e.g. head orientation or eye gaze), that can be used by a perceiving individual to predict a sender's subsequent behaviour (e.g. based on where another individual is looking) (Freeberg *et al*., 2021). The production of cues was likely selected for their direct benefits to the sender rather than for their influence on a receiver.

Like human communication, studies of non‐human animal communication have focused on signals and been carried out largely within a sender–signal–receiver framework. One popular model is the code model (Cartmill, 2023) according to which signals are encoded by a signaller, transmitted *via* a communicative channel, and then received and decoded by a receiver (Rendall, Owren & Ryan, 2009; Scott‐Phillips, 2015). Human communication is thought to differ from non‐human communication in that it relies not only on signal en‐ and decoding, but also involves the provision and interpretation of communicative and informative intentions (Scott‐Phillips, 2015; Crivelli & Fridlund, 2018) as for example in the ostensive‐inferential model (Sperber & Wilson, 1986).

Many researchers argue for continuity in the evolution of human communication from non‐human communication (Pinker & Bloom, 1990; Arbib, 2005) and that our understanding of human processes could benefit from comparative research into non‐human primates, our closest living relatives. However, such comparative research has traditionally studied gestures, vocal and facial behaviours separately (Slocombe, Waller & Liebal, 2011) and resulted in a problematic modality dichotomy (Tomasello, 2008; Liebal & Oña, 2018). Within this dichotomy, gestures are viewed as intentionally produced signals and contrasted with vocalizations and facial expressions which, due to a greater relevance for emotion signalling, are thought to be more difficult or impossible to control (Tomasello, 2008). Accordingly, non‐human animal emotions have been tackled largely in the vocal and facial modalities with insights being often indirect and dependent on inferences from accompanying behaviours or the situational context (Bard, 2003).

There is now an increasing awareness that non‐human primate communication should be studied using a multimodal approach (Slocombe *et al*., 2011; Liebal, Carpenter & Tomasello, 2013) and that more aspects including temporal communication dynamics must be considered (Schirmer, Meck & Penney, 2016; Krumhuber *et al*., 2023). These dynamics not only shape the significance of cues and signals, but also influence a receiver's response. A relevant phenomenon in this context is chorusing, which refers to the temporal coordination or synchronization of interacting conspecifics. Chorusing has been observed across a wide range of species including fireflies (Buck & Buck, 1968), frogs (Ryan, Tuttle & Taft, 1981), and gibbons (Raimondi *et al*., 2023). It has been linked to a range of functions like mate attraction and bonding (Schirmer *et al*., 2016; Kotz, Ravignani & Fitch, 2018; Ravignani, 2019) and is believed to be the evolutionary precursor of human rhythmic coordination in the context of music and dance. Indeed, like many other species, humans have a tendency to mirror the non‐verbal behaviours of interaction partners and to align temporally their physiological and motor rhythms, especially when interaction partners are likable or bonded (Hoehl, Fairhurst & Schirmer, 2021).

In sum, non‐human communication is thought to involve signals and cues. Whereas signals evolved for communicative, perceiver‐mediated effects, cues evolved not for communication but for their direct benefit to senders. Nevertheless, cues can be communicative as they may influence a perceiver's behaviour. Because communication is a multimodal, dynamic process, signals and cues should be studied in their sensory and temporal complexity.

## VI. SITUATING CUES AND SIGNALS IN THE CONTEXT OF HUMAN EMOTIONS

Events that can elicit emotions come in various forms. In the context of communication, they may be usefully categorized into non‐social and social events. Non‐social events include, among others, startling noises, delicious foods, and foul odours. They induce responses such as stiffening, mouth watering, or nose scrunching, which serve in bodily protection, energy consumption, and disease prevention, respectively. Social events are encounters with other individuals of the same or another species, for example in the context of predation, mating, or parenting. The emotions they evoke are often considered to be social emotions (e.g. anger, pride). They depend on communication (Parkinson, 2021), are socially constructed, and are thus inherently shared between interactants (Boiger & Mesquita, 2012; Barrett, 2017; Parkinson, 2021).

We reason that non‐social events are more likely than social events to trigger non‐verbal changes that can be defined as cues. These events likely evoke emotions that, in the absence of a social context, must rarely be regulated and prompt behaviours to deal with these events in a self‐beneficial way. Bystanders may accurately infer emotions with only limited information such as an agent's face. Social events, by contrast may be more likely than non‐social events to trigger non‐verbal changes that can be defined as signals. As they concern another person to which the individual is already attending, they promote communication and increase the possibility of other‐mediated benefits. Moreover, social non‐verbal changes may be partially or entirely regulated due to the interactants' goals and social norms as well as the interactional dynamics. They may be shaped by the responses of interaction partners and emerging temporal patterns. Perhaps because of this, “recognizing” the emotions evoked by social events may require more than non‐verbal cues and signals and depend on context.

In short, the concept of signals and cues derived from non‐human research can be mapped onto situations in which humans communicate their emotions. It necessitates that that we dissociate emotion communication for non‐social and social events and highlights the complexity created by the latter.

## VII. NON‐VERBAL EFFECTING

Insights from non‐human communication and emotion research prompt us to argue for a broader perspective on human NVEE. While the classic sender–signal–receiver model of information transfer is clearly valuable, we also recognize a need to expand this model and to move beyond the current focus on facial expressions and social emotions. For this, we consider it useful to re‐frame our thinking by adopting some of the terms and ideas developed in work on non‐human species.

**Fig. 5:**
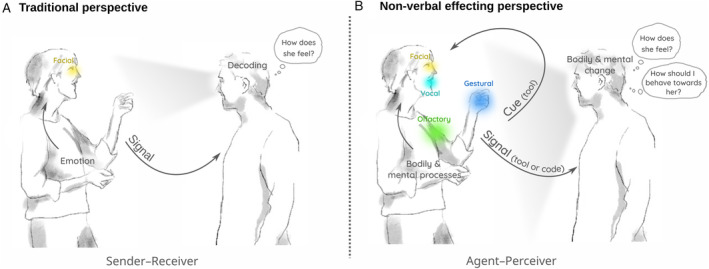
Non‐verbal communication of emotions in human interactions. (A) In traditional perspectives, senders express their emotions through non‐verbal messages, while receivers decode these messages in order to understand how a sender feels. Such studies have focused largely on the face. (B) Non‐verbal effecting holds that an agent's emotions to a social or non‐social event trigger non‐verbal responses multimodally. These responses may benefit the agent directly (e.g. increased visual field) and indirectly through processes triggered in a perceiver (e.g. increased liking of the agent, mental inferences about an agent's state or motives). Responses with only direct benefits are referred to as cues and those with (additional) indirect benefits as signals. Both cues and signals may allow perceivers to infer an agent's state and predict their behaviour. However, they may also change the perceiver's own emotions and thus affect how they themselves feel, think and behave. To understand the significance of a non‐verbal response it must be examined in its multi‐modal context and with reference to its effects for both agent and perceiver. Note that this figure shows a social event. Non‐social events would be expected to produce cues only.

We propose that human NVEE involves both cues and signals (Fig. 5). Although both terms are currently used in the human literature, their meaning is not consistently differentiated, thus biasing notions that all non‐verbal expressions have a communicative function for the sender. Dissociating cues and signals allows us formally to repudiate this notion and to begin decoupling sender and receiver processes. We suggest further that, alongside a possible use as codes, non‐verbal behaviours are also considered a tool of influence. It is indisputable that, for example, facial expressions or hand gestures can be referential and convey a particular meaning. Yet, it is unlikely that their effect on interaction partners is strictly due to such a meaning. How someone moves, sounds, or smells also has an immediate influence that can, for example, attract or repel another. To accommodate this influence, we suggest focusing on the *effect* of NVEEs and to refer to mechanisms (codes/tools) only when those can be clearly specified. Finally, we caution against using the terms “sender” and “receiver” as they are deeply rooted in the traditional perspective and thus have unwanted theoretical implications. Instead, we prefer the terms “agent” and “perceiver”, which accommodate better the ideas of cues, signals, and effects.

This broadened perspective opens new methodological approaches that promise important new insights. This includes expanding our limited focus on the explicit recognition of NVEEs by considering all individuals involved and their interactional dynamics. Initial steps include studying the agent and how his or her non‐verbal behaviours may be directly self‐beneficial. An example is provided by research showing that eyebrow raising, as seen in fear, increases an agent's visual field thus promoting threat detection and that nose scrunching, as seen in disgust, decreases olfactory intake thus reducing contamination risk (Susskind *et al*., 2008). These effects on the agent suggest that aspects of fear‐ and disgust‐related non‐verbal behaviours may have evolved as cues, without a communicative purpose. Indeed, examining non‐verbal responses to emotional events in the absence of perceivers and pursuing their impact on an agent's perceptual, cognitive and/or motor function might enable further cue discoveries.

Next, however, it will be important to consider agent and perceiver as an interacting unit and to explore NVEE differences between isolated and interactional settings as a means to probe cue *versus* signalling functions (e.g. signals may more readily emerge in interactional settings; interactional settings may help elucidate indirect/mediated benefits for the agent). Additionally, the synchrony that unfolds between agent and perceiver will be relevant. Their dynamics may modify or augment the “meaning” of individual non‐verbal behaviours. Exemplary evidence for this comes from research showing that mirroring or temporal alignment with a perceiver can modulate an agent's emotional arousal (Murata *et al*., 2021), contribute to the perceiver's understanding of the agent's feeling state (Wood *et al*., 2021), and enhance cooperation and joint performance (Gordon *et al*., 2020; Behrens *et al*., 2020).

A second important direction will be to move beyond the face and to study the modalities in combination. Past efforts have typically focused on individual channels. However, cortical representations of the world are multisensory (Ghazanfar & Schroeder, 2006) including representations that entail social interactions. As detailed above, non‐verbal behaviours are by nature multimodal and their importance in the context of human evolution (Dunbar & Shultz, 2007; Hari *et al*., 2015) implies that their perception must be multimodal too. Indeed, there is increasing evidence that conceptually dividing the senses is flawed. Some percepts mix at the level of receptors (e.g. chemical and temperature sensing on the skin) (Caterina *et al*., 1997; Peier *et al*., 2002; Ackerley *et al*., 2014), while others converge at higher levels in the brain (Ghazanfar & Schroeder, 2006). Studying them in combination may show that limitations in emotion recognition in one modality are reduced or absent if perceivers have access to all modalities (Bänziger *et al*., 2009; Cao *et al*., 2014; Fernández Carbonell, Boman & Laukka, 2021). Accordingly, an anxious body odour, for example, facilitates the recognition of fear in faces with raised eyebrows and wide eyes (Silva *et al*., 2020).

As mentioned above, a multimodal approach has already gained traction within the non‐human animal literature (Partan & Marler, 1999; Partan, 2017; Liebal, Slocombe & Waller, 2022) where, for example, antipredatory defence has been considered with respect to different communication channels (Kikuchi *et al*., 2023). Although the need for a multimodal approach to human communication has been articulated (e.g. Gregori *et al*., 2023), there are as yet but few published attempts to look at more than two modalities concurrently (Bänziger *et al*., 2012; Monroy, Cowen & Keltner, 2022). That adoption of multimodality in empirical research remains slow is likely due to other methodological considerations. Indeed, whereas non‐human non‐verbal behaviours are often explored in fairly naturalistic settings (Shutt *et al*., 2007; Soares *et al*., 2011; Schirmer, Seow & Penney, 2013*b*; Dennis, Shuster & Slobodchikoff, 2020), the wish to control extraneous variables leads human researchers to create artificial paradigms focusing on the mental and bodily responses of perceivers to primarily static facial displays. Indeed, a recent meta‐analysis examining brain responses to both positive and negative social stimuli highlighted the fusiform gyrus, a brain region that forms part of the visual processing pathway and is relevant for the perception of faces (Atzil *et al*., 2023). In the same analysis, responses of auditory, somatosensory and olfactory regions were not reported, underscoring a strong visual bias in research. To correct this bias, human research will have to revise its core paradigms.

Finally, we believe it is time to move beyond tasks that require participants to recognize emotions. It is more important to explore the effects of non‐verbal behaviours on perceivers as these effects may map better than verbal labels on how emotions are communicated (Crivelli & Fridlund, 2018). Two types of effects are of interest. On the one hand are effects on a perceiver's ability to predict the agent's attitudes and behaviours. On the other hand, a tool‐like influence on the perceiver should be measurable as a change in bodily, mental or behavioural processes (Van Kleef, De Dreu & Manstead, 2006; Parkinson & Simons, 2009; van Kleef, 2014; Crivelli *et al*., 2015; Mehu & Scherer, 2015; Cohen‐Chen *et al*., 2022). One study examining the former effects on perceiver predictions asked participants to indicate for a given facial expression the nature of the appeal (e.g. to help, to stop, to celebrate) that an agent directed at a perceiver (Scarantino *et al*., 2022). Different emotions were associated with different core appeals (e.g. happiness → to celebrate or affiliate). A study examining the latter, tool‐like effects presented participants with neutral words spoken with a sad or neutral tone (Schirmer *et al*., 2013*a*, p. 200). Relative to the neutral tone, the sad tone elicited greater attention‐related neural responses in the electroencephalogram and this effect was associated with better memory for word content and an affective decrease in how word content was evaluated. Although participants could not remember a word's tone of voice, they later evaluated words more negatively when they had heard them with a sad as compared with a neutral tone. Thus, these data imply interesting immediate and delayed effects of non‐verbal signals on mental and bodily processes that may ultimately bias perceiver behaviours so as to benefit the agent.

Summing up, we suggest a non‐verbal effecting perspective, which holds that human agents produce emotional cues and signals that can influence perceivers without needing to be decoded. To understand NVEE, we must investigate the effects of cues and signals for human agents and perceivers in natural, multimodal settings.

## VIII. IMPLICATIONS AND DIRECTIONS FOR RESEARCH

We believe that a non‐verbal effecting perspective could have profound consequences for our understanding of human NVEEs and the human mind more generally. It accommodates the possibility that we can discover emotional “meaning” in an agent's cues and signals. Considering an agent's behaviour multimodally, including its underlying physiology, provides a better chance of identifying associated feeling states that may map onto an emotion or emotion cluster (e.g. social affiliative states). Moreover, dissociating between cues and signals as well as their non‐social and social effects promises novel insights into the functions of NVEEs and their relationship to human well‐being. Indeed, the role of social interactions and non‐verbal communication is now increasingly recognized as central to most mental health issues (Brown *et al*., 1997; Derntl *et al*., 2011). Thus, exploring these issues in terms of the effects of non‐verbal behaviours on both agents and perceivers could usefully contribute to understanding and re‐conceptualizating clinical disorders in light of the Research Domain Criteria (RDoC) project initiated by the National Institute of Mental Health (Insel *et al*., 2010; Cuthbert, 2020).

There is as yet little work on the effects of human non‐verbal communication that considers the complexity associated with the interacting individuals, modalities, and consequences. We have argued here that such research is sorely needed but that concerns about experimental control produce biases in favour of simplistic and artificial study designs. Yet, technical, computational, and statistical advances now help address these concerns. They offer tools that can tackle complex data patterns to derive meaningful insights. Thus, instead of presenting faces for emotion recognition, two or more interacting individuals can be studied concurrently in emotionally provoking situations such as a competitive or cooperative task. Non‐verbal (Künecke *et al*., 2014; Tschacher, Rees & Ramseyer, 2014; Drimalla *et al*., 2019; Schirmer, Lo & Wijaya, 2021) as well as other bodily processes (Ayrolles *et al*., 2021; Reinero, Dikker & Van Bavel, 2021) can be recorded *via* hyper‐scanning and the relationships examined between these data, emotions, and task performance. The unpredictability of real interactions and the multidimensionality of the resultant data may be daunting. However, novel software solutions now make analysis approaches such as cross‐correlations, Granger causality, and multivariate statistics easily accessible. Moreover, some of these approaches readily deal with data in which information unfolds dynamically across time. Especially promising in this regard, is a multivariate technique called representational similarity analysis (RSA) (Kriegeskorte, Mur & Bandettini, 2008; Popal, Wang & Olson, 2020). Its key advantage is the possibility to compare and relate different data types such as the NVEEs of an agent and the task performance of a perceiver along multiple data features or dimensions.

The fact that our understanding of how humans communicate emotions non‐verbally is at present still limited, biased by faces, and likely flawed (Barrett *et al*., 2019) creates real‐life issues. One example is that we are unable to provide adequate support to individuals with sensory or other deficits that impede on the ability to produce and/or perceive non‐verbal cues and signals. To address such deficits as well as the non‐verbal needs of the public more generally, engineers have looked to develop artificial intelligence (AI) solutions for emotion recognition and responding. Like the non‐verbal literature, this development has been biased towards faces and the idea that facial behaviours reveal underlying emotion categories. A literature search of the AI‐related research similar to that shown in Fig. 4 (see Appendix S1) confirmed that faces were the most popular modality (ranked 13/100 themes) with the other modalities not featuring among the 100 most frequent themes. While still in its early stages, such research has already been commercialized (e.g. *iMotions*, *TAWNY*, *Noldus*) and is now available for anyone wanting to “know” how others feel. Current applications include health care but also market research, and human–robot interactions (Khare *et al*., 2024), extending increasingly into other domains such as work and organizational settings (Boyd & Andalibi, 2023; Roemmich, Schaub & Andalibi, 2023). Against this backdrop, the need to revisit the scientific approach to NVEE and to educate the public accordingly is ever more pressing.

Taken together, a non‐verbal effecting perspective opens up new possibilities not only in the context of communication research but for understanding the human condition more generally. Novel tools are now available to tackle associated data analytic challenges and should be used to further our understanding of NVEE and to correct faulty popular notions that have problematic real‐world consequences.

## IX. CONCLUSIONS

(1) Like other species, humans communicate their emotions non‐verbally.
(2) Although research has made considerable progress in elucidating this process, the search for a non‐verbal code has been only partially successful. Moreover, questions have been raised as to how human NVEE should be conceptualized and studied.
(3) To address these questions, the perspective developed here applies insights and ideas from the study of non‐human animals. (*i*) It emphasizes the importance of considering both agents and perceivers and the dynamic manner in which their interaction unfolds. (*ii*) It seeks to extend the current investigation of faces to other non‐verbal channels and to study non‐verbal exchanges holistically, in real‐life interactions. (*iii*) Its focus on the effects of non‐verbal behaviours complements traditional ideas about information transfer with ideas that agents employ tools to benefit themselves directly (cues) or indirectly, *via* changes in an interaction partner (signals).
(4) The present perspective, referred to as “non‐verbal effecting”, thus promotes a broader, less human‐centric approach to how our species communicates emotions. It holds that, to understand behaviours such as winking and smiling, we must pursue their function together with other concurrent non‐verbal behaviours for both agent and perceiver.

## Supporting information


**Appendix S1.** Details of literature search procedure and analysis.
