# Supplementary material for: Non‐verbal effecting – animal research sheds light on human emotion communication
Source: Biol Rev Camb Philos Soc. 2024 Sep 11;100(1):245–57. doi: 10.1111/brv.13140 (PMC11718621; doi:10.1111/brv.13140)
Supplement: Supplementary file 1 — Appendix S1. Details of literature search procedure and analysis. [file BRV-100-245-s001.docx]

**Appendix S1.** Details of literature search procedure and analysis.

**Literature search**

We conducted a literature search using the *rscopus* package (Muschelli, 2022) in R (R Core Team, 2015). To identify studies on humans, we queried published articles with both ‘emotion’ or ‘affect’ and ‘nonverbal’ or ‘non-verbal’ in the title, abstract or key words. To identify work in non-human animals, we had to modify our search term as ‘nonverbal’ was irrelevant. Thus, we queried articles with ‘emotion’ or ‘affect’, ‘communication’ and ‘social’, and ‘nonhuman’, ‘non-human’, or ‘animal’ in the title, abstract or key words. Our specific query for humans was “TITLE-ABS-KEY (emotion* OR affect*) AND TITLE-ABS-KEY (nonverbal OR non-verbal) AND NOT TITLE-ABS-KEY (artificial AND intelligence)”, whereas for non-human research it was “TITLE-ABS-KEY (emotion* OR affect*) TITLE-ABS-KEY (communication AND social) AND TITLE-ABS-KEY (nonhuman OR non-human OR animal)”.

We retrieved 10,800 human and 2317 non-human abstracts. A subsequent search for artificial intelligence (AI)-related research used the terms “(TITLE-ABS-KEY (emotion* OR affect*) AND TITLE-ABS-KEY (recognition) AND TITLE-ABS-KEY (artificial intelligence)” and returned 4215 abstracts.

**Literature analysis**

We subjected the retrieved abstracts to a text-mining routine using the *tokenizer* package in R (Mullen *et al.*, 2018). We first removed hyphens so that hyphenated words (e.g. non-verbal → nonverbal) would not be split into separate tokens (e.g. ‘non’ and ‘verbal’). After the tokenizing, we removed tokens that were redundant (e.g. nonverbal, found, data, communication, stimuli). The same tokens were removed from the human and the non-human data. Next we removed stop words and pronouns keeping only adjectives, adverbs, nouns, and verbs. We then counted the frequency of each unique lemma in the human and the non-human data and computed a proportion by dividing each computed frequency by the respective maximum frequency thus accounting for differences in the number of human and non-human abstracts. Note that lemmas helped us combine different tokens/wordforms with the same meaning (e.g. signalling, signal). Fig. 4 shows the lemma proportions for each species.
